# The combination model of CNN and GCN for machine fault diagnosis

**DOI:** 10.1371/journal.pone.0292381

**Published:** 2023-10-05

**Authors:** Qianqian Zhang, Caiyun Hao, Zhongwei Lv, Qiuxia Fan

**Affiliations:** School of Automation and Software Engineering, Shanxi University, Taiyuan, P.R. China; Seoul National University of Science & Technology, REPUBLIC OF KOREA

## Abstract

Learning powerful discriminative features is the key for machine fault diagnosis. Most existing methods based on convolutional neural network (CNN) have achieved promising results. However, they primarily focus on global features derived from sample signals and fail to explicitly mine relationships between signals. In contrast, graph convolutional network (GCN) is able to efficiently mine data relationships by taking graph data with topological structure as input, making them highly effective for feature representation in non-Euclidean space. In this article, to make good use of the advantages of CNN and GCN, we propose a graph attentional convolutional neural network (GACNN) for effective intelligent fault diagnosis, which includes two subnetworks of fully CNN and GCN to extract the multilevel features information, and uses Efficient Channel Attention (ECA) attention mechanism to reduce information loss. Extensive experiments on three datasets show that our framework improves the representation ability of features and fault diagnosis performance, and achieves competitive accuracy against other approaches. And the results show that GACNN can achieve superior performance even under a strong background noise environment.

## Introduction

Industries are the basis of the nation’s economy. In modern industries, smart factories employ smart manufacturing, and the basic building block of any manufacturing process industry is the machines [1, 2]. Mechanical equipment is often subject to extreme and complex working conditions, thus significantly increasing the possibility of failure. Once any failure occurs in machines, it will not only reduce the production efficiency and cause economic losses, but also threaten the safety of human production and work. In order to avoid these economic and safety risks, health state monitoring and evaluation systems for types of machinery have been widely developed and implemented in modern industries [3–7].

Nowadays, with the development of intelligent method, it has been used in the current fault diagnosis of the machine widely. Different from model-based and traditional data driven based models which require priori knowledge and expert experience, deep learning (DL) based on models with better ability to capture features by deep networks has brought new ideas for machine fault diagnosis [8–11]. Zhou et al. [12] proposed a new improved multi- scale edge-labeling graph neural network (MEGNN) to enhance the recognition accuracy of DL-based TCM under small samples.

Among these studies, convolutional neural network (CNN), as one of the most commonly used DL methods, has also been widely studied and applied in this field. Focus on the small sample problem, Dong et al. [13] proposed a new intelligent fault diagnosis framework based on dynamic model and transfer learning for rolling element bearings race faults. Su et al. [14] proposed a convolutional neural network based on hierarchical branches for faults diagnosis, which made three predictions from coarse to precise through three hierarchical branches, and the model has good diagnostic performance in noisy environments and variable working conditions. Pang et al. [15] have reported a deep coupled based autoencoder neural network wherein microphone and accelerometer data fusion was implemented to detect the defects in gear orbit and bearing. As the most commonly used deep learning model, convolutional neural network (CNN) can better learn feature representations, more and more scholars at home and abroad introduce it into fault diagnosis of mechanical equipment. For instance, Kumar et al. [16] proposed a novel convolutional neural network (NCNN) based on small sample data for bearing defect recognition. Aiming at the low efficiency of CNN, He et al. [17] proposed a new inverted residual convolutional neural network, which can improve the diagnostic efficiency while ensuring the accuracy. In the feature mining module, a one-dimensional Convolutional Neural Network (1-D CNN) is utilized to extract features from raw vibration signals. An et al. [18] proposed a domain adaptation network based on contrastive learning (DACL) to achieve the aim of bearing fault diagnosis cross different working conditions and reduce the probability of samples being classified near or on the boundary of each class to improve diagnosis accuracy. Cao et al. [19] proposed an unsupervised domain-share convolutional neural network for efficient fault transfer diagnosis of machines from steady speed to time-varying speed. Mao W et al. [20] proposed a new deep auto-encoder method with fusing discriminant information about multiple fault types, the proposed method can effectively improve the diagnostic accuracy with acceptable time efficiency.

In order to enhance the feature extraction capability of the network, the attention mechanism is also integrated into the neural network applied to machine fault diagnosis, which can make the network focus on important information and reduce information loss, such as Debasish Jana et al. [21] proposed a two-step framework based on CNN and convolution auto encoder (CAE) based on real-time sensor fault detection, localization, and correction, which effectively improved the generalization ability of the model.

Although the abovementioned iterative neural network model based on the backpropagation supervised learning method has achieved considerable results, the CNN model still has considerable limitations. The convolution based on Euclidean data is difficult to grasp the complex topological structure. Graph convolutional networks (GCNs) taking graph data with topological structure as input is more efficient for data relationship mining, making GCN to be powerful for feature representation from graph data in non-Euclidean space. Zhou et al. [22] constructed a dynamic graph data processing framework for rotating machinery diagnosis. Wang et al. [23] proposed a multiple micrographs-based graph convolutional network for surface defect detection using an image dataset. Li et al. [24] developed a multi-receptive field GCN-based fault diagnosis model using an imbalanced dataset, which not only features from different receptive fields, but also fuses learned features as an enhanced feature representation.

Both CNN and GCN are deep learning methods that can capture deep features, whether it is CNN or GCN, the network constructed by only a single convolutional layer has limited ability to capture information, which is the limitation of the receptive field. For GCN, the network learns on a predefined single graph structure, which can only aggregate the information of the nearest neighbor nodes.

On the basis of previous work, to further improve the feature representation ability and fault diagnosis performance, this study proposes a novel GACNN, which combines the aggregation of global and spatial features through CNN and GCN to improve feature representation capabilities. Specifically, the GCN branch takes the complete graph as input, and the CNN branch takes the node feature of the complete graph as input. Additionally, and uses ECA attention mechanism to reduce information loss.

The main contributions of this work are summarized as:

1. A novel fault diagnosis framework based on GACNN is proposed, which integrates the strengths of CNN and GCN to extract global and spatial features. And a ECA attention mechanism is introduced to reduce information loss.
2. The relationships between input signals are explicitly measured by converting the raw signal into a complete graph, which effectively mines the relationship between the structural features of a sample.
3. The detailed parameter analysis is carried out for GACNN. Three public experimental datasets of the bearing system verified the effectiveness of the proposed method and algorithm.

The rest of this paper is organized as follows. Section 2 is mainly about the basic theoretical model for GCN and CNN. In section 3, the mechanical fault diagnosis pipeline with GACNN is illustrated in detail. Section 4 presents some comparative experiments and analysis to prove the excellent performance of the proposed model. In Section 5, it will draw the conclusion and prospect for the future research.

## Theoretical background

### Convolutional neural network

**Fig 1:**
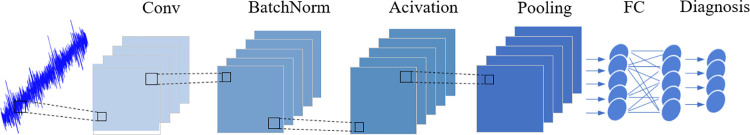
CNN for fault diagnosis.

CNN aims to learn abstract features by alternating and stacking convolutional layers and pooling layers. In the convolutional layers, multiple local filters convolve with raw input data and generate translation-invariant local features. The subsequent pooling layers extract features with a fixed length over sliding windows of the raw input data by following several rules such as average, max and so on. The general CNN model in fault diagnosis is shown in Fig 1.

In signal processing, 1D-CNN is utilized to calculate delay accumulation of signals with the same kernel. The forward propagation of the convolution layer is expressed as:

$$h_{j}=ReLu(\sum_{i=1}^{c}x_{i}*w_{ij}+b_{j})$$ (1)

Where *x*_*i*_ is the *i*-th channel of the input feature mapping. *h*_*j*_ is the *j*-th channel of the output feature mapping, *w*_*ij*_ and *b*_*j*_ are the convolution kernel weights and biases, respectively, and * represents the convolution operation. *ReLu*(∙) is the activation function.

To improve the training process, Batch Normalization (BN), a training optimization method is utilized to achieve normalization and standardization of batch data during training. BN can stabilize the distribution of the input data, prevent overfitting, and accelerate the training process. Therefore, by integrating pooling and BN layers, CNN can effectively extract discriminative features from the input data.

### Graph convolutional network

Graph convolutional network-based method can effectively mine relationship between nodes in the graph by feature aggregation and transformation. For the undirected graphs *G* = (*V*,*ξ*,*A*) [25], where *V* = *n* represents the finite nodes. The *ξ* is a set of edges and *A*∈*R*^*n*×*n*^ denotes the adjacency matrix of graph *G*. For two nodes <*v*_*i*_,*v*_*j*_> in a graph, the value of *A*_*ij*_can be denoted as:

$$A_{ij}=\{\begin{array}{l} 1,\;while\;the\;node<v_{i},v_{j}>is\;connected\\ 0,\;while\;the\;node<v_{i},v_{j}>is\;not\;connected \end{array}$$ (2)

As a kind of data in the non-Euclidean domain, graphs are represented by adjacency matrices. Usually, the symmetric normalized graph Laplacian will be used, which is defined as:

$$L=D^{-1/2}LD^{-1/2}=I_{N}-D^{-1/2}AD^{-1/2}$$ (3)

Where *L*∈*R*^*n*×*n*^ denotes the Laplacian matrix, *L*_*N*_∈*R*^*n*×*n*^ is the identity matrix. *D*∈*R*^*n*×*n*^ is the diagonal degree matrix of the graph with *D*_*ii*_ =∑_*j*_*A*_*ij*_.

The spectral graph convolution of the node *v* with the node features can be defined as:

$$h={\left(x{*}_{G}f\right)}_{\theta }=U(U^{T}xU^{T}f)$$ (4)

Where *x* is the node feature, *h* represents the feature maps after graph convolution. *f* is the eigenfunction of Λ, i.e. *f*(Λ), *θ* is the learnable parameter, *G stands for graph convolution.

Consider *f*_*θ*_ = *U*^*T*^*f* as the learnable graph convolution filter, then the above formula is simplified to:

$$h={\left(x{*}_{G}f\right)}_{\theta }=U(U^{T}x(U^{T}f))=Uf_{\theta }U^{T}x$$ (5)

**Fig 2:**
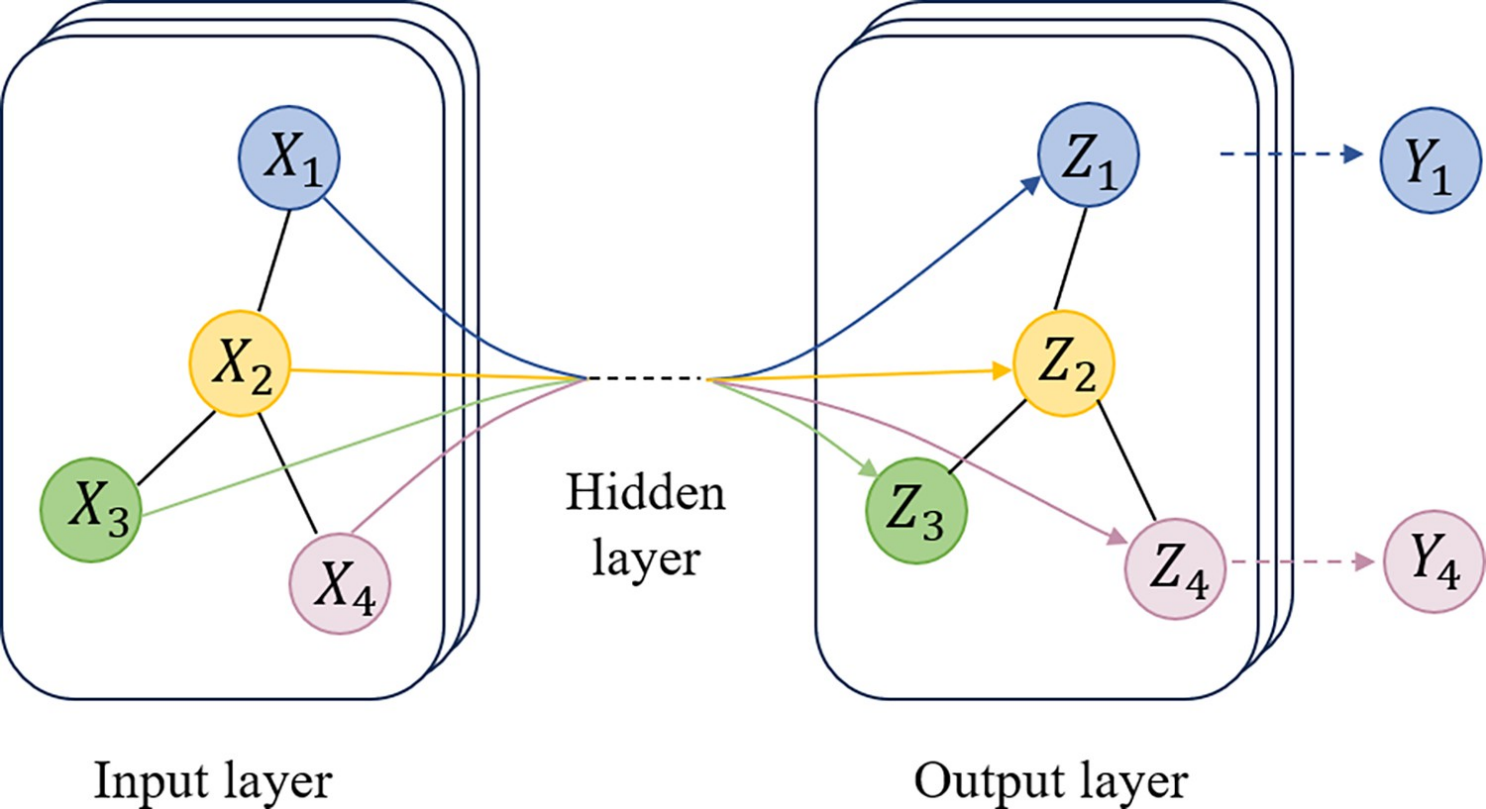
Typical graph convolution network architecture.

Where *U*^*T*^*x* represents the graph Fourier transform of the node feature *x*. The standard two-layer GCN model shown in Fig 2.

The computational complexity of graph convolution shown in Eq (5) is very large, and it is not localized in space. In GCN, the filter *f*_*θ*_ is approximated by a polynomial, and it is suggested in [26] that the truncated expansion of the Chebyshev polynomials can approximate *f*_*θ*_ very well, and the *K*^*th*^ order approximation is:

$$f_{\theta^{\prime }}=\sum_{k=0}^{K-1}\theta_{k}\Lambda^{k}=\sum_{k=0}^{K-1}\theta_{k}^{\prime }T_{k}(\tilde{\Lambda })$$ (6)

Where *K* is the maximum order of Chebyshev polynomials, $\tilde{\Lambda }=2\Lambda /\lambda_{\max}-I_{N}$ is the rescaled eigenvalues, *λ*_max_ denotes the largest eigenvalues of *L*. *θ* is a vector of polynomial coefficients, $\theta_{k}^{\prime }\in R^{K}$ is the vector of Chebyshev coefficients. $T_{k}(\tilde{\Lambda })$ is the Chebyshev polynomial of order *k*, and it can be determined by the following recurrence relation:

$$\{\begin{array}{l} T_{k}(x)=2xT_{k-1}(x)-T_{k-2}(x)\\ T_{0}(x)=1,T_{1}(x)=x \end{array}$$ (7)

After approximating the filter by Chebyshev polynomials, the graph convolution of a node feature *x* with a filter *f*_*θ*_ in spectral domain can be mathematically defined as follows:

$$h=\sum_{k=0}^{K-1}\theta_{k}^{\prime }T_{k}(\tilde{L})x$$ (8)

Where $\tilde{L}=2L/\lambda_{\max}-I_{N}$ is the rescaled Laplacian matrix. A parameterized weight matrix *W*∈*R*^*S*×*M*^ is adopted to perform matrix transformation and achieve feature transformation. The output of GCN layer *X*′∈*R*^*N*×*M*^ is:

$$X^{\prime }=Cheb(X,W)=Wh$$ (9)

Where *Cheb*(⋅) is the Chebyshev graph convolution.

## Methods

**Fig 3:**
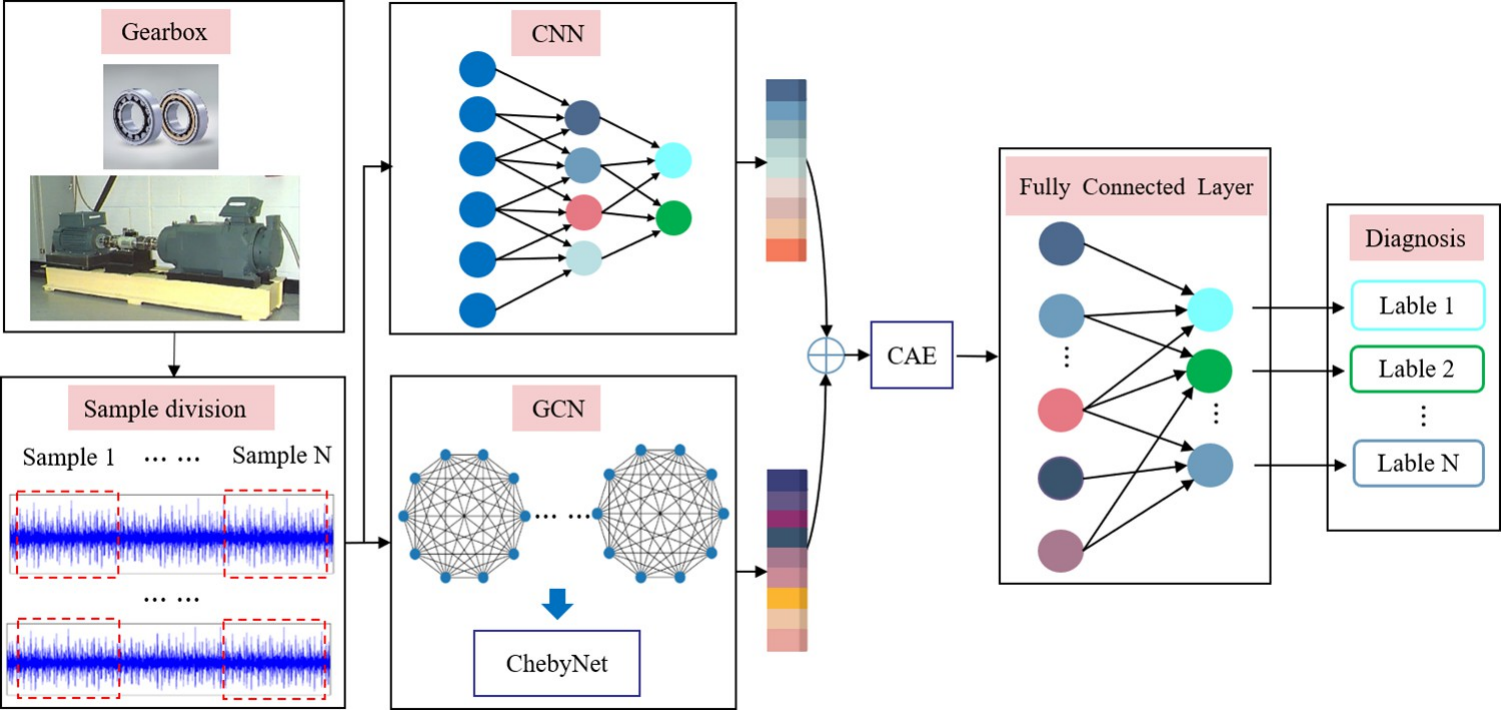
Structure of the GACNN.

This section details how to apply the proposed GACNN for mechanical fault diagnosis, the process involves CNN-based branch、GCN-based branch and Efficient Channel Attention with GACNN. The overall flowchart of the proposed method for mechanical fault diagnosis is shown in Fig 3, and it summarizes as the following algorithm.

**Algorithm: GACNN for mechanical fault diagnosis**

**A. The complete graph construction**

**Input**: the raw signal *X* with length *L*, select the normalize methods, set the sub-sample length *d*, set the number of nodes *q* in complete graph.

**Output**: the complete graph dataset *H*_*train*_,*H*_*test*_;

1. Obtain the normalized signal: $X^{nor}=(x_{1}^{nor},x_{2}^{nor},\cdots ,x_{m}^{nor})$;

2. Obtain the spectrum amplitude features: *Y*^*nor*^ = *FFT*(*X*^*nor*^);

3. Data split: M, *n* = *floor*(*L*/*d*)←*Y*^*nor*^;

4. Constructing a complete graph:

 for subset X in M

  for *i* = 1,2,⋯,*q* do

   for *j* = 1,2,⋯,*q* and *j*≠*i* do

        $w_{ij}=\exp(-\frac{{\left\|\left(x_{i},x_{j}\right)\right\|}^{2}}{2\zeta^{2}}),x_{j}\in Ne(x_{i})$

   end for

  end for

 end for

5. Obtain complete graph set: *G*_*train*_,*G*_*test*_

6. Obtain training set and test set: *H*_*train*_,*H*_*test*_.

   **B. Fault diagnosis with GACNN**

**Input**: *H*_*train*_,*H*_*test*_.

**Output**: the fault category *Z*

1. Model training:

2. for *X* in *H*_*train*_ do

  *Z*←*GACNN*(*X*)

  $CE\leftarrow -\sum_{i=1}^{c}y_{i}\log(p_{c})$

  Update with back propagation

 end for

3. Fault diagnosis and classification: *Z*←*GACNN*(*H*_*test*_)

### CNN-based branch

The CNN-based branch network is modified from the simple and effective CNN model. The convolutional layers are assembled within three convolution blocks, each convolutional block includes a convolutional layer, a normalization layer, a pooling layer, and an activation layer. The max pooling layer retains the maximum value of the region and preserves the features with high recognition, which can reduce the error caused by the convolutional layer parameters. The size of feature output of convolution block is defined as *h*×*w*×*d*, where *h* and *w* are spatial dimensions and *d* is the number of channels.

The CNN-based branch is firstly leveraged to capture features from input data, and the extract feature maps can be denoted as:

$$h_{1}=CNN(X_{input})$$ (10)

### GCN-based branch

In our work, GCN is a novel neural network that learns feature by gradually aggregating information in the neighborhood. GCN directly operates on a graph, and outputs the embedding vector of nodes according to the nature of the neighborhood of the node. First, the signal sample can be modeled by graphs, the nodes of which stand for the detected objects and the edges of which represent distance between the nodes. Then, we use three-layer Chebyshev graph convolution operation to capture the dependencies among sample graphs. Finally, a dropout layer is added to prevent the network model from overfitting.

**Fig 4:**
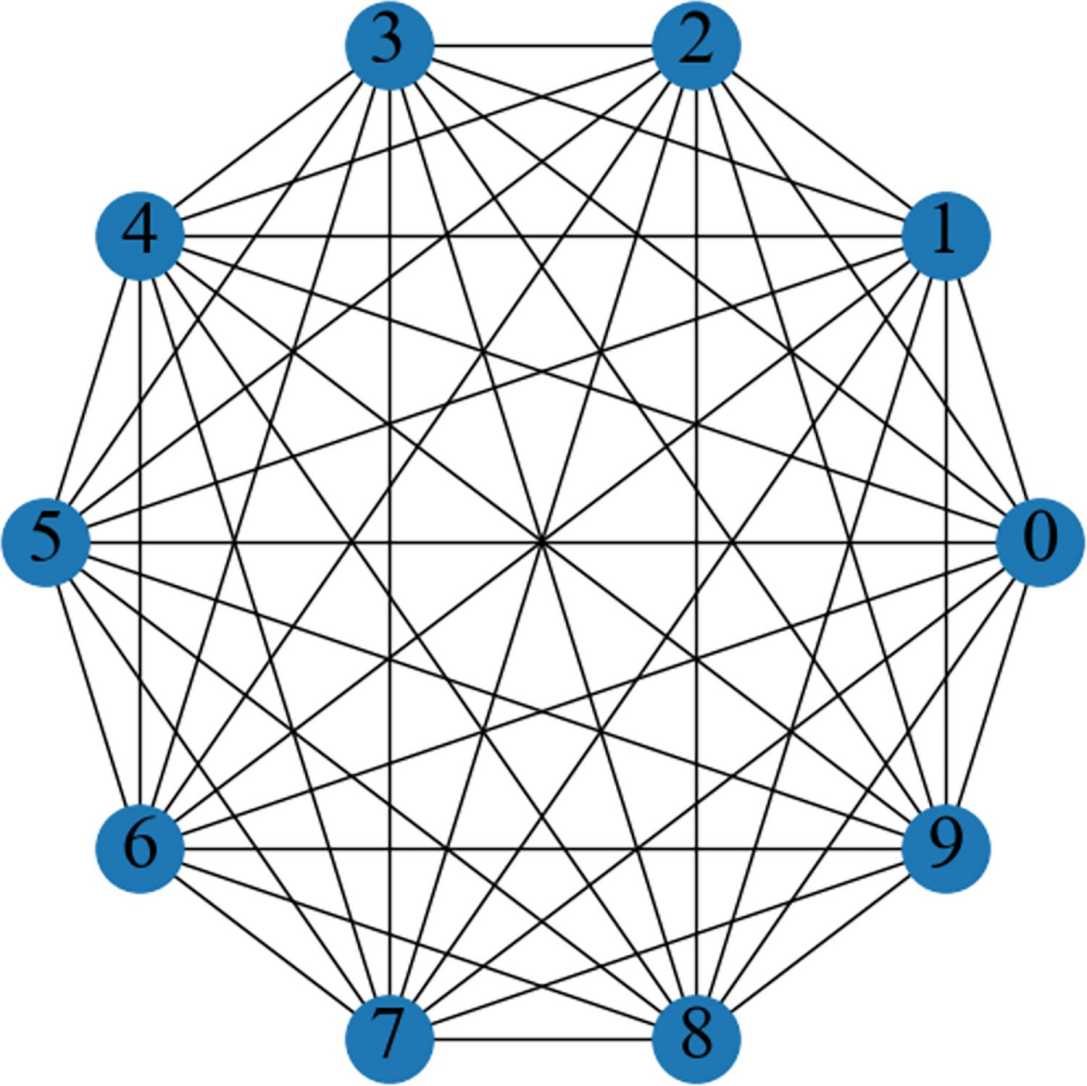
Example of graph construction using complete graph.

As the input to GCN, we need to build the vibration signal into a graph structure. Therefore, a complete graph is developed. As shown in Fig 4, a complete graph means that any two nodes have an edge connected between them, that is, the nodes are connected by pairs. In a complete graph, each pair of different vertices is connected by exactly one edge. The edge weight between each node of the complete graph can be estimated by a Gaussian kernel weight function, which is defined as:

$$w_{ij}=\exp(-\frac{{\left\|\left(x_{i},x_{j}\right)\right\|}^{2}}{2\zeta^{2}}),x_{j}\in Ne(x_{i})$$ (11)

Where ‖(*x*_*i*_,*x*_*j*_)‖^2^ represents the calculation of the Euclidean distance between nodes. *w*_*ij*_ denotes the edge weight between node *x*_*i*_ and node *x*_*j*_. *ζ* is the bandwidth of the Gaussian kernel, *ζ* = 0.01.

The GCN-based branch is leveraged to capture features from input graph data, and the extract feature can be denoted as:

$$h_{2}=GCN(X_{input})$$ (12)

### Efficient channel attention

**Fig 5:**
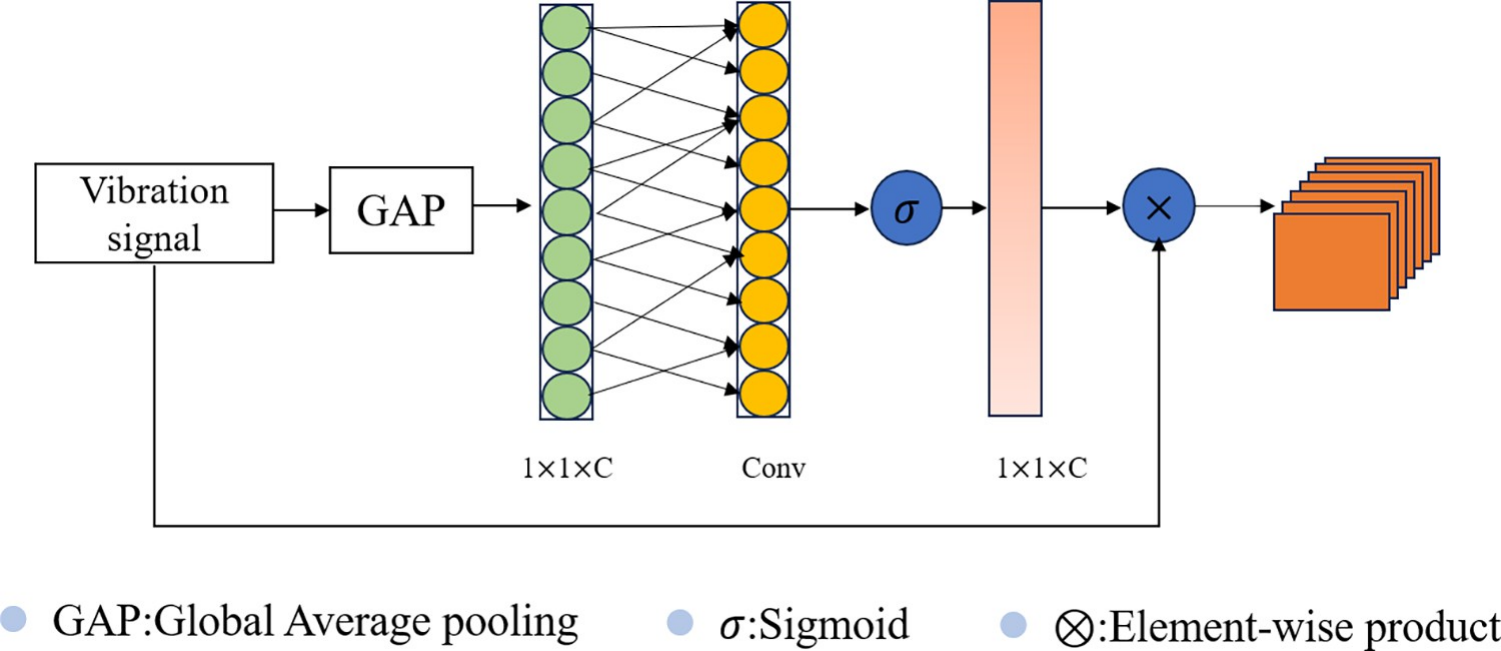
The architecture of the attention block.

ECA attention module can enhance the channel features of input features, and avoids the impact of dimensionality reduction on data by means of one-dimensional convolution, and does not change the size of input features. Despite CNN-based branch and GCN-based branch can extract global and spatial features, in these multi-scale features, there are important features with obvious differences, as well as noncorrelated features that are difficult to distinguish. In order to improve the contribution of relevant features and reduce the interference of invalid features on fault diagnosis results, the 1D-signal Efficient Channel Attention (ECA) attention mechanism module is designed to reduce information loss and increases anti-noisy ability, as shown in Fig 5 [27]. After the ECA module uses global average pooling (GAP) aggregation convolution features without dimensionality reduction, then performs one-dimensional convolution and performs sigmoid function learning.

### Feature fusion and classification

After completing the two-stage convolution through CNN-based branch and GCN-based branch, we splice the features, *h*_1_ and *h*_2_, obtained by the two branches in the channel dimension for further fusion in subsequent modules.

$$H_{out}=ECA(h_{1}⊕h_{2})$$ (13)

Where ⊕ denotes the concatenation operation. *h*_1_ and *h*_2_ represents the information extracted by CNN-based branch and GCN-based branch, respectively. *ECA*(⋅) represents ECA attention mechanism module.

Finally, the fusion of features extracted from the two branches is delivered to the classifier to make the final prediction, node classification is processed by fully connected network as follows:

$$Z=soft\max(FC(H_{out}))$$ (14)

Where Z is the predicted label.

Usually the cross-entropy (CE) loss would be used for classification, and the CE loss over all labeled nodes can mathematically denoted as:

$$CE=-\sum_{i=1}^{c}y_{i}\log(p_{c})$$ (15)

Where *c* is the number of categories. The *y*_*i*_∈(*y*_1_,*y*_2_,⋯,*y*_*c*_) is the set of node labels, and *y*_*i*_ is the indicator variable (0 or 1). The *p*_*c*_ is the predicted probability that the observation sample belongs to category *c*. The weight parameters of GACNN layer can be updated, through back propagation algorithm.

### Data enhancement

In this study, the original vibration signal will be transformed into 1-D spectrogram. Data normalization is operated on the monitoring signal *X* = (*x*_1_,*x*_2_,⋯,*x*_*m*_) and the normalized signal $X^{nor}=(x_{1}^{nor},x_{2}^{nor},\cdots ,x_{m}^{nor})$ can be calculated as:

$$x_{i}^{nor}=\frac{x_{i}-x_{\min}}{x_{\max}-x_{\min}},i=1,2,\cdots ,m$$ (16)

Then, fast Fourier transform (FFT) is performed on the normalized signal *X*^*nor*^ to obtain the spectrum amplitude as the features, which can be denoted as:

$$Y^{nor}=FFT(X^{nor}),i=1,2,\cdots ,m$$ (17)

Where FFT(⋅) is the Fast Fourier transform, *Y*^*nor*^ = (*y*_1_,*y*_2_,⋯,*y*_*m*_) is the spectrum amplitude of *X*^*nor*^, and the half of result are taken as the node features *f* = (*y*_1_,*y*_2_,⋯,*y*_*m*/2_).

## Experimental validation and discussion

Experiments were conducted on three public datasets, to verify the effectiveness of the proposed method. In addition, parameter selection of the experiments was performed to obtain the optimal model parameter. In order to verify the anti-noise performance of this method in mechanical fault diagnosis, we tested the anti-noise performance on the basis of case 2 dataset.

All the models trained with 100 epochs, an initial learning rate of 0.001, and the Adam optimizer. The accuracy of test samples in the table represents the average value of five experimental results, and serves as the final experimental result. The main framework was developed using Python.

In order to demonstrate the efficiency and superiority of the proposed method, comparison with other DL methods is essential. Methods for comparison contain CNN [28], Chebyshev graph convolution network [29], and multi-receptive field GCN (MRF-GCN) [30]. Details of above these methods is shown in Table 1.

**Table 1:** Setting of the models of the bearing dataset.

Model	Parameter configuration
**GACNN**	Input size:320*1024, structure of GCN:1024—512—output_dim.
**MRF_GCN**	Input size:320*1024, structure of GCN:1024–1200–300.
	*K* = 1,2,3. fc: 300—output_dim.
**ChebyNet_3**	Input size:320*1024, structure of GCN:1024—512—300—output_dim, *K* = 3.
**CNN**	Input size:320*1*1024, structure of CNN:320*1*1024–320*8*512–320*16*255–32*126. fc: 512—output_dim

### Case1: CWRU bearing dataset

#### Data description

**Fig 6:**
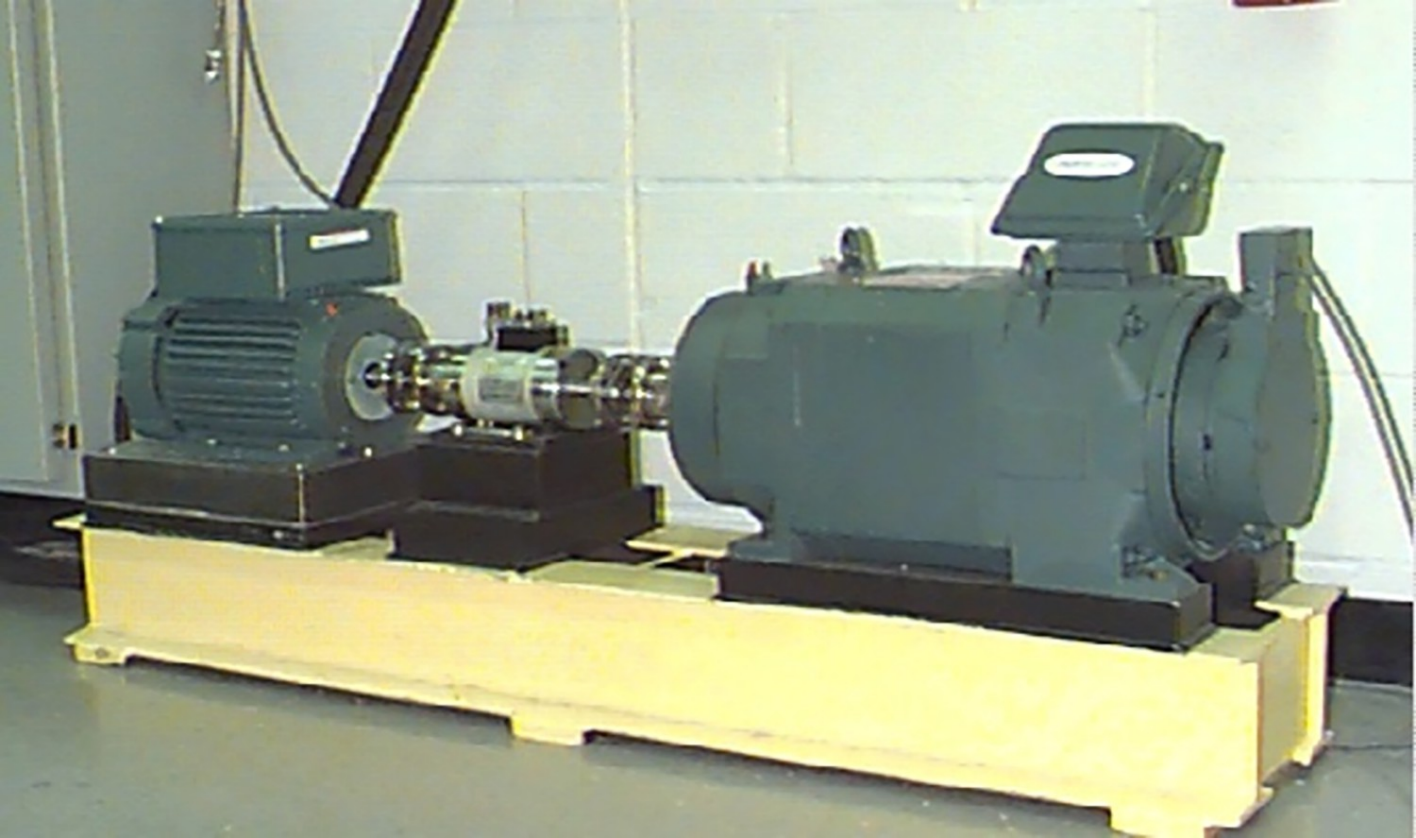
Experimental platform used by CWRU.

In this study, the public dataset is provided by Case Western Reserve University (CWRU) is utilized to evaluate the diagnostic validity and generalization capability of the proposed approach [31]. The test rig of CWRU comprises a 1492-W test motor, a torque transducer/encoder, a dynamometer, and a load motor, as depicted in Fig 6. The bearing conditions include a healthy condition (H) and three fault conditions, namely inner race fault (IF), outer race fault (OF), and ball fault (BF). The fault diameter for each fault condition is 7 mils, 14 mils, and 21 mils, respectively.

To construct the experimental dataset, we selected the bearing vibration signal under the condition of an acceleration sensor sampling frequency of 48 kHz, motor load of 0 hp, 1 hp, 2 hp, and 3 hp. The resulting dataset contained 800 samples, each comprising four different sensor signals with a sample length of 1024 data points. To divide the dataset into training and testing sets, 60% of the samples in each health state were randomly selected as the training set, and the remaining 40% were considered as the testing set. Table 2 shows the results of the dataset production.

**Table 2:** The description of class labels of CWRU.

Load	Condition	Fault size (mils)	Label
**0/1/2/3**	NA	0	0
	IF	7	1
	IF	4	2
	IF	21	3
	OF	7	4
	OF	4	5
	OF	21	6
	BF	7	7
	BF	4	8
	BF	21	9

#### Parameter selection

**Fig 7:**
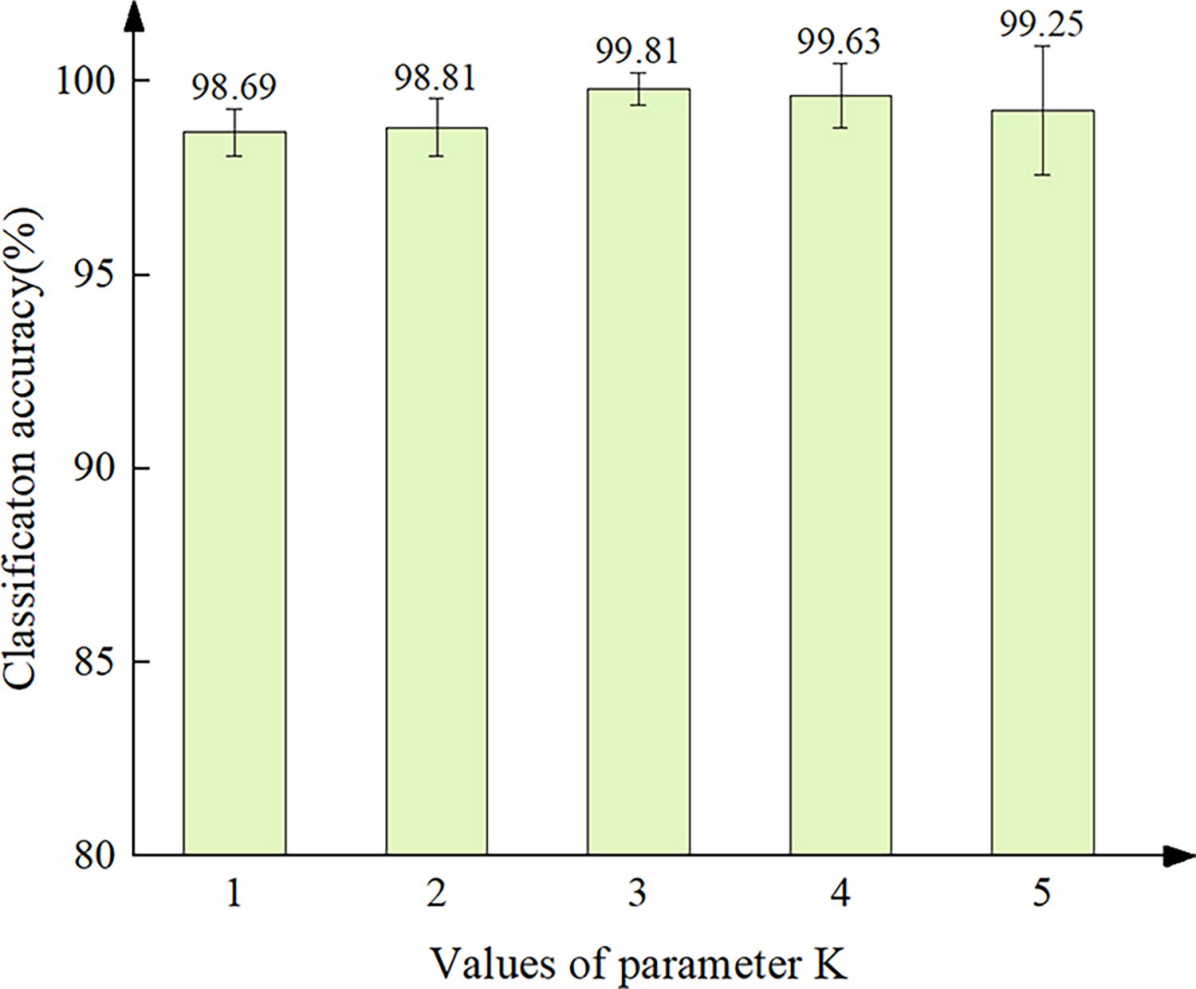
Experiment result for selection of parameter *K*.

The parameter *K* is a crucial parameter in determining the performance of GCN, as it determines the order of the Chebyshev polynomial and, consequently, the number of hops of the neighboring nodes that are aggregated during graph convolution. By selecting an optimal value of *K*, the GCN can effectively aggregate information from similar nodes during the convolution process. Therefore, in this study, we conducted experiments to investigate the effect of varying *K* on classification accuracy. Specifically, we evaluated the classification accuracy for *K* values ranging from 1 to 5 and obtained the average results of five experiments, as shown in Fig 7.

From the results presented in Fig 7, it can be show that the classification accuracy increased when *K* increased from 1 to 3, but decreased when *K* increased from 3 to 5. The optimal value of *K* was found to be 3, resulting in a classification accuracy of 99.81%. Consequently, *K* = 3 was selected as the optimal parameter for our subsequent experiments.

#### Discussion

The results of the comparison are presented in Table 3. It can be observed from those results that the proposed GACNN has the highest fault diagnosis accuracy, and the stability of GACNN is better than other methods. The accuracies of GACNN in each trial are 98.12%、100%、99.06%、100%、100%, respectively, with a standard deviation of 0.84%, which demonstrating that GACNN can provide more greater accuracy. The reason for this superior performance is that GACNN can aggregate global and spatial features and enhance the feature learning ability.

**Table 3:** The diagnostic result of CWRU.

Models	Max-acc (%)	Min-acc (%)	Avg_acc (%)
**GACNN**	100	98.12	99.44±0.84
**MRF_GCN**	97.50	93.75	95.25±1.92
**ChebyNet_3**	96.88	93.12	94.56±1.43
**CNN**	99.69	95.31	97.31±1.83

### Case2: MFPT bearing dataset

#### Data description

The Society for Machinery Failure Prevention Technology (MFPT) dataset is composed of four sets of bearing vibration signals, including a baseline dataset, seven outer race fault datasets, seven inner race fault datasets, and some other datasets [32]. In this study, the former three datasets are used, therefore, it can be considered as a 15-class classification task. And the results of the dataset production are shown in the Table 4.

**Table 4:** The description of class labels of MFPT.

Fault location	Condition	Label
baseline dataset	Baseline_1	0
**outer race fault datasets**	Outer Race Fault_vload_1	1
	Outer Race Fault_vload_2	2
	Outer Race Fault_vload_3	3
	Outer Race Fault_vload_4	4
	Outer Race Fault_vload_5	5
	Outer Race Fault_vload_6	6
	Outer Race Fault_vload_7	7
**inner race fault datasets**	Inner Race Fault_vload_1	8
	Inner Race Fault_vload_2	9
	Inner Race Fault_vload_3	10
	Inner Race Fault_vload_4	11
	Inner Race Fault_vload_5	12
	Inner Race Fault_vload_6	13
	Inner Race Fault_vload_7	14

**Fig 8:**
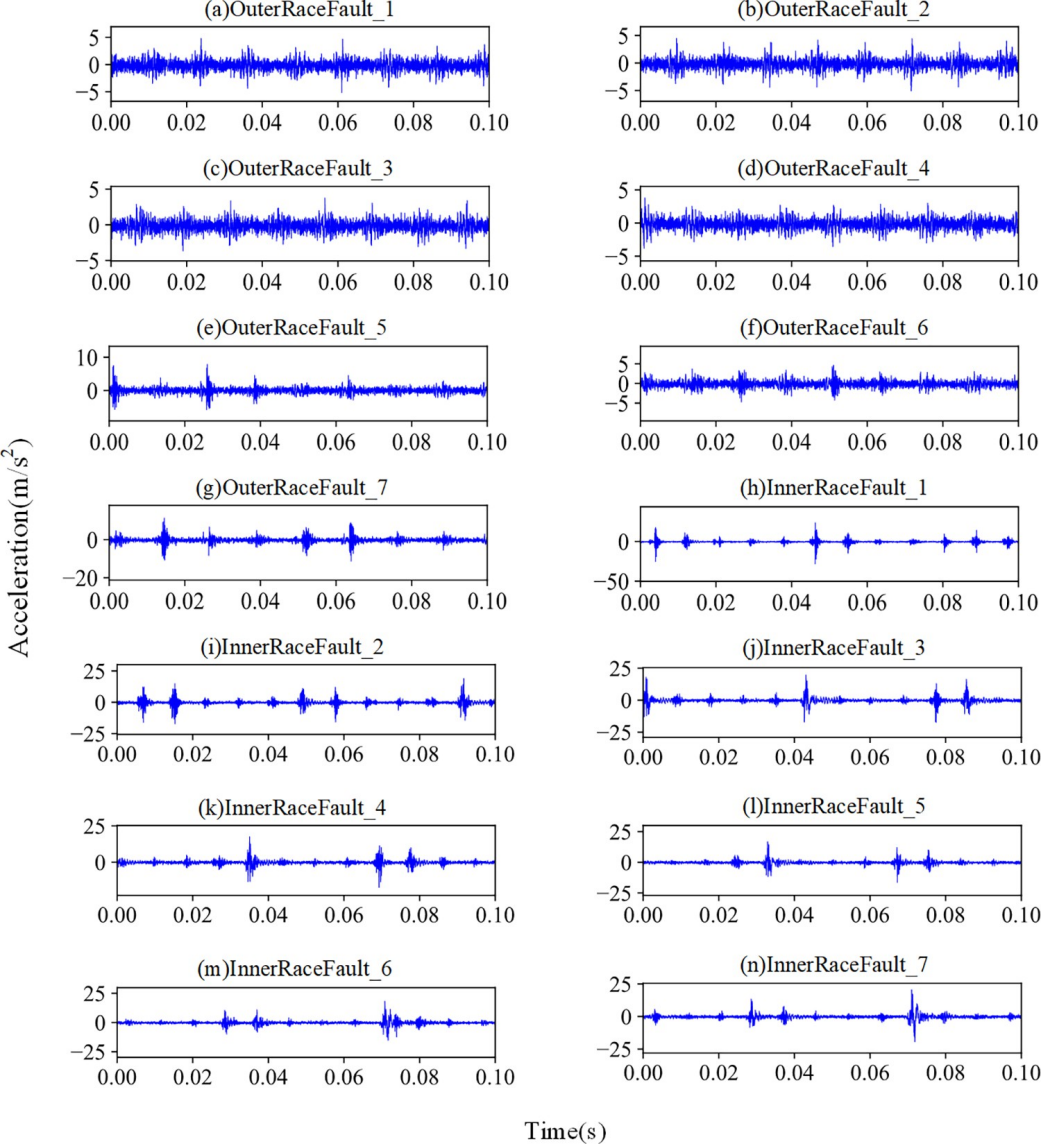
Time domain output of fault samples in MFPT dataset.

In this experiment, the number of data point in a sample is set to 1024. There are 50 samples for each load, resulting in a total of 750 samples in the bearing dataset. To divide the dataset into training and testing sets, 60% of the samples in each health state were randomly selected as the training set, and the remaining 40% were used for testing. Time domain diagrams of fault samples with all states are given in Fig 8.

#### Discussion

The experimental results are shown in Table 5. It can be observed from those results that the diagnosis accuracies are quite similar between the four kinds of mothed, however, the proposed GACNN has the highest fault diagnosis average accuracy. The accuracies of GACNN in each trail are 97.41%, 97.78%, 96.67%, 97.04%, 96.67%, respectively, confirming the effectiveness of the proposed method. Furthermore, it is shown in Table 5 that the avg-acc of GACNN is 97.11%, and the standard deviation is 0.48%. The standard deviation of GACNN is the smallest one among four methods, which verifies its superior stability.

**Table 5:** The diagnostic result of MFPT.

Models	Max-acc (%)	Min-acc (%)	Avg_acc (%)
**GACNN**	97.78	96.67	97.11±0.48
**MRF_GCN**	97.67	96.00	96.80±0.65
**ChebyNet_3**	97.33	96.33	96.80±0.38
**CNN**	92.00	90.67	91.27±0.49

### Case3: SEU bearing dataset

#### Data description

Southeast University (SEU) gearbox datasets were provided by Southeast University. The dataset contains a gear dataset and a bearing dataset, which were both acquired by Drivetrain Dynamic Simulator (DDS) [33]. In this study, the bearing dataset is selected for the test, each state is considered as a type, so it becomes a 10-class classification task. And the results of the dataset production are shown in the Table 6.

**Table 6:** The description of class labels of SEU.

Component	Condition	Label
**Bearing Fault**	Health_20_0	1
	Healthl_30_2	2
	Ball_20_0	3
	Ball_30_2	4
	Inner_20_0	5
	Inner_30_2	6
	Outer_20_0	7
	Outer_30_2	8
	Comb_20_0	9
	Comb_30_2	10

In this experiment, the number of data point in a sample is set to 1024. There are 50 samples for each fault condition, thus a total of 500 samples were contained in the bearing dataset. 60% of the samples in each health state were randomly selected as the training set, and the other 40% were regarded as the testing set.

#### Discussion

Average accuracy of five trials is used to analyze, and the results are shown in the Table 7. Compared to CNN and GACNN, GACNN obtains the highest classification accuracy, reaching 95.00%, the avg-acc of GACNN is 92.80%, and the standard deviation is 1.60%, verifying effectiveness of the proposed method. GCNs taking graph data with topological structure as input is more efficient for data relationship mining, enriching the feature learning ability of the GACNN. Therefore, GACNN can obtain highest classification accuracy among these methods.

**Table 7:** The diagnostic result of SEU.

Models	Max-acc (%)	Min-acc (%)	Avg_acc (%)
**GACNN**	95.00	91.50	92.80±1.60
**MRF_GCN**	95.00	90.00	91.90±1.95
**ChebyNet_3**	93.00	89.50	90.80±1.44
**CNN**	90.50	86.00	88.70±2.25

### Robustness of the GACNN

Machinery equipment are often exposed to a noisy working environment in the real world. In order to verify the robustness of the proposed method under a strong background noise environment, Gaussian white noise was performed on the original vibration signal from the MFPT dataset of case 2 to simulate the real environment. The Signal-to-Noise Ratio (*SNR*) was used to measure the noise intensity, and its expression is as follows:

$$SNR=10\log(\frac{P_{signal}}{P_{noise}})$$ (18)

Where *P*_*signal*_ and *P*_*noise*_ are signal energy and noise energy, respectively. *SNR* represent the noise intensity in *dB*.

In this paper, Gaussian noise was performed on the original vibration signal with noise intensity ranging from −5*dB* to 10*dB*. The vibration signals containing noise were used as training datasets and test datasets to simulate the real strong background noise environment. The training datasets and test datasets were generated randomly each time to reduce the randomness of the experimental results.

**Fig 9:**
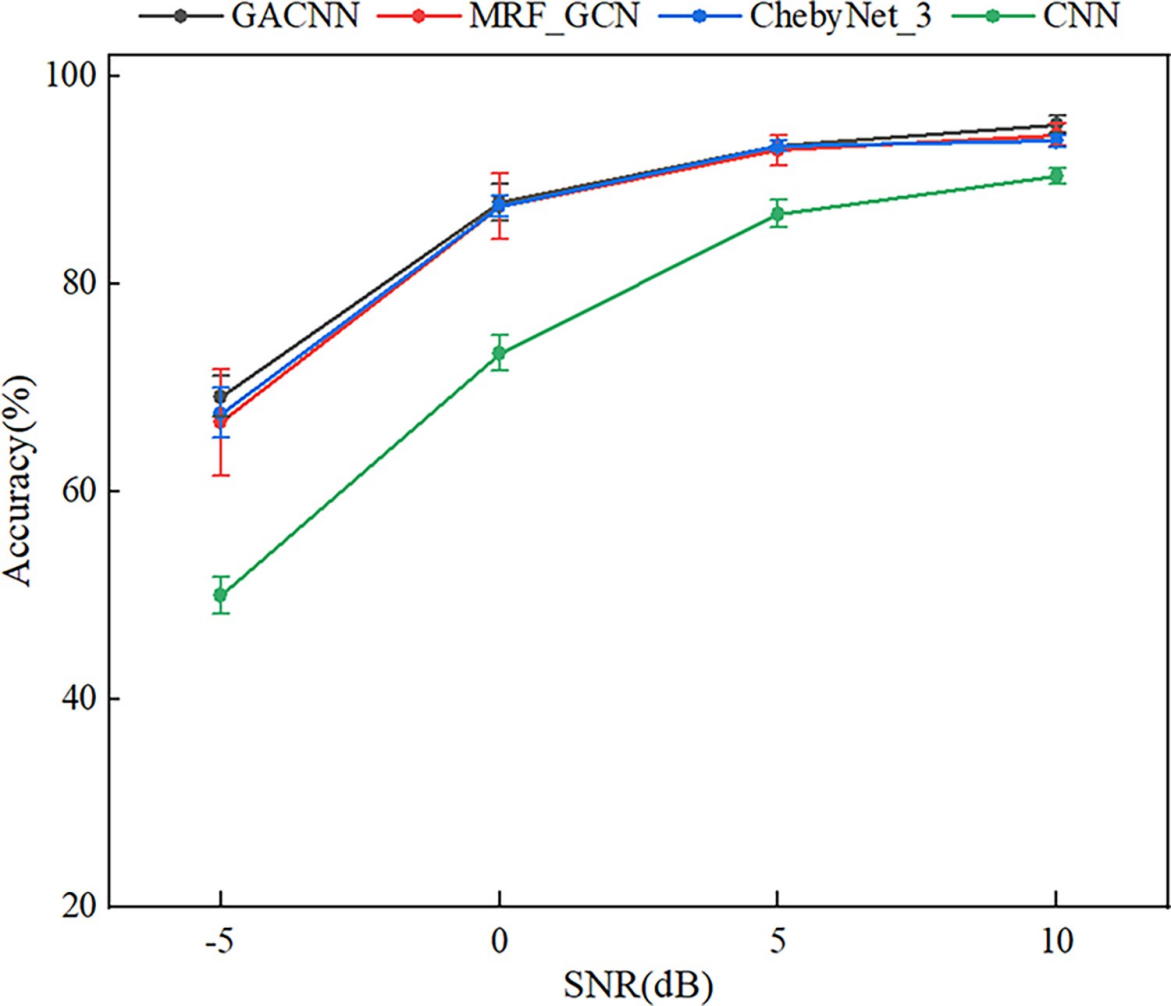
The experimental results of anti-noise performance.

To ensure the accuracy and consistency of the experimental results, the five trials were conducted in this study to eliminate the effects of randomness. The experimental result under different noise level is shown in Table 8 and Fig 9. It can be observed that the performance of GACNN decreases with the increase of noise, and increases with the decrease of noise. Specifically, under a strong background noise environment, the proposed GACNN exhibited the highest fault diagnosis accuracy among all methods. These findings suggest that the GACNN is capable of accurately diagnosing faults even in challenging real-world conditions, making it a promising approach for practical applications in fault diagnosis.

**Table 8:** Experiment results of MFPT datasets under different noise level.

SNR Models	10	5	0	-5
**GACNN**	95.33	93.27	87.80	69.07
**MRF_GCN**	94.33	92.87	87.47	66.67
**ChebyNet_3**	93.73	93.19	87.46	67.53
**CNN**	90.33	86.73	73.33	50.00

## Conclusions

In this paper, a novel fault diagnosis framework was proposed that utilizes the strengths of both CNNs and GCNs to extract both global and spatial features. To ensure the intelligence and improve the diagnosis efficiency and accuracy, the vibration signals in frequency domain are used to form samples without manual feature extraction, and the frequency domain signal and the fully connected diagram are used as the input of the fault diagnosis framework. The performance of our proposed method in the fault diagnosis of rotating machinery is comprehensively evaluated through three experimental cases. The conclusions can be summarized as follows:

(1) A novel fault diagnosis framework based on GACNN is proposed, which integrates the strengths of CNN and GCN to extract global and spatial features.

(2) Comprehensive experiments are conducted on three open-source datasets to evaluate the performance of proposed method. The diagnostic accuracy on the three datasets was 99.44%, 97.11% and 92.80%, respectively, which achieved highest classification accuracy compared with the comparison model

(3) In the MFPT dataset, the anti-noise performance experiment is carried out, which verifies the robust performance of the proposed method under strong background noise environments.

In addition, while the framework represents a significant improvement over existing technologies, there are the following limitations and opportunities for future research that should be addressed: (1) The actual industrial field data can be noisier and more complex, it was only simulated by adding noise. Therefore, further validation of the real industrial data is still needed; (2) The category distribution of real industrial data was often unbalanced, namely, the number of normal data was far more than the amount of faulty data, but it is assumed that the number of categories samples are equal.
